# Development and validation of glycolysis‐related prognostic score for prediction of prognosis and chemosensitivity of pancreatic ductal adenocarcinoma

**DOI:** 10.1111/jcmm.16573

**Published:** 2021-05-03

**Authors:** Xiu‐Ping Zhang, Qinjunjie Chen, Qu Liu, Yang Wang, Fei Wang, Zhi‐Ming Zhao, Guo‐Dong Zhao, Wan Yee Lau, Yu‐Zhen Gao, Rong Liu

**Affiliations:** ^1^ Faculty of Hepato‐Biliary‐Pancreatic Surgery Chinese People’s Liberation Army (PLA) General Hospital Beijing China; ^2^ Department of Hepatic Surgery IV The Eastern Hepatobiliary Surgery Hospital Second Military Medical University Shanghai China; ^3^ Faculty of Medicine The Chinese University of Hong Kong Hong Kong China; ^4^ Department of Clinical Laboratory Sir Run Run Shaw Hospital Zhejiang University School of Medicine Hangzhou China

**Keywords:** chemosensitivity, glycolysis, pancreatic ductal adenocarcinoma, prognosis, score

## Abstract

Pancreatic ductal adenocarcinoma (PDAC) is a lethal malignancy with aggressive biological behaviour. Its rapid proliferation and tumour growth require reprogramming of glucose metabolism or the Warburg effect. However, the association between glycolysis‐related genes with clinical features and prognosis of PDAC is still unknown. Here, we used the meta‐analysis to correlate the hazard ratios (HR) of 106 glycolysis genes from MSigDB by the cox proportional hazards regression analysis in 6 clinical data sets of PDAC patients to form a training cohort, and a single group of PDAC patients from the TCGA, ICGC, Arrayexpress and GEO databases to form the validation cohort. Then, a glycolysis‐related prognosis (GRP) score based on 29 glycolysis prognostic genes was established in 757 PDAC patients from the training composite cohort and validated in 267 ICGC‐CA validation cohort (all *P* < .05). In addition, including PADC, the prognostic value was also confirmed in other 7 out of 30 pan‐cancer cohorts. The GRP score was significantly related to specific metabolism pathways, immune genes and immune cells in the patients with PADC (all *P* < .05). Finally, by combining with immune cells, the GRP score also well‐predicted the chemosensitivity of patients with PADC in the TCGA cohort (AUC = 0.709). In conclusion, this study developed a GRP score for patients with PDAC in predicting prognosis and chemosensitivity for PDAC.

## 1 INTRODUCTION

Pancreatic ductal adenocarcinoma (PDAC) is a lethal malignancy with a dismal 5‐year survival rate of 9%.^1^ It ranks fourth amongst all causes of cancer‐associated deaths in the world.^2^, ^3^ The dismal prognosis is related to late diagnoses and limited effectiveness of systemic treatments. PDAC is a cancer with no significant improvements made in diagnosis and therapy in the past 30 years. Radical resection with negative margins, (R0 resection), is the only key to long‐term survival for this aggressive tumour.^4^ Despite considerable progresses made in understanding this disease at the molecular level, novel findings have yet been translated into clinical benefits, and most patients still face a grim median survival of 5‐6 months.^5^ Whether the molecular findings can be translated into clinical use by integrating mutation genes of PDAC to establish a score in predicting clinical prognosis and guide treatments need further studies.

Recently, increasing evidence suggests that reprogramming of tumour metabolism as novel therapeutic targets can be used as an effective anticancer strategy.^6^ A high rate of aerobic glycolysis, known as the Warburg effect, is a hallmark of cancer cell glucose metabolism.^7^ Recent studies reported that aerobic glycolysis is active in PDAC in promoting pancreatic tumorigenesis, proliferation and invasion.^8^, ^9^, ^10^ Furthermore, pyruvate kinase M2, which promotes cell survival and invasion under metabolic stress by enhancing the Warburg effect in PDAC,^11^ promotes PDAC invasion and metastasis through phosphorylation and stabilization of PAK2 protein.^12^ Also, TWIST1, which transcriptionally regulates glycolytic genes, promotes Warburg metabolism in pancreatic cancer.^9^ In addition, oncogenic Kras driven metabolic reprogramming in pancreas cancer cells utilizes cytokines from tumour microenvironment,^13^ and tumour‐associated macrophages in tumour microenvironment promote progression and the Warburg effect via the CCL18/NF‐kB/VCAM‐1 pathway in PDAC.^14^ By transforming growth factor beta‐induced protein, which is an extracellular matrix interacting protein, glycolysis is enhanced and pancreatic cancer cell migration promoted.^15^ Only limited studies exist which systematically investigated the metabolic status and its prognostic value in patients with PDAC. To clarify the relationship between glycolysis and PDAC is crucial in better understanding the mechanism of tumorigenesis and in predicting prognosis of patients in different risk groups.

A previous study^16^ indicated that a six‐gene risk signature related to glycolysis could predict survival outcomes in patients with hepatocellular carcinoma (HCC), and high‐risk scores were associated with unfavourable survival outcomes. This study provided novel insights into the relationship between glycolysis and HCC. Another study^17^ identified consistently dysregulated genes within the glucose metabolic pathways. On investigating the prognostic power of these genes on survival outcomes in HCC patients, two distinct molecular HCC subtypes were identified, with one subtype having significantly worse prognosis. Again such findings provided novel mechanistic and clinical insights for development of personalized management of HCC patients. The association between genetic characteristics of glycolysis and heterogeneity of PDAC has rarely been reported.

In this study, a glycolysis‐related prognostic signature was developed from the whole genome expression data for patients with PDAC coming from several data sets. The study aimed to find out whether this prognostic signature could be used to detect a group of patients with PDAC with high risks of unfavourable survival outcomes and to identify PDAC with different degrees of chemosensitivity.

## 2 MATERIALS AND METHODS

### 2.1 PDAC patients

**FIGURE 1:**
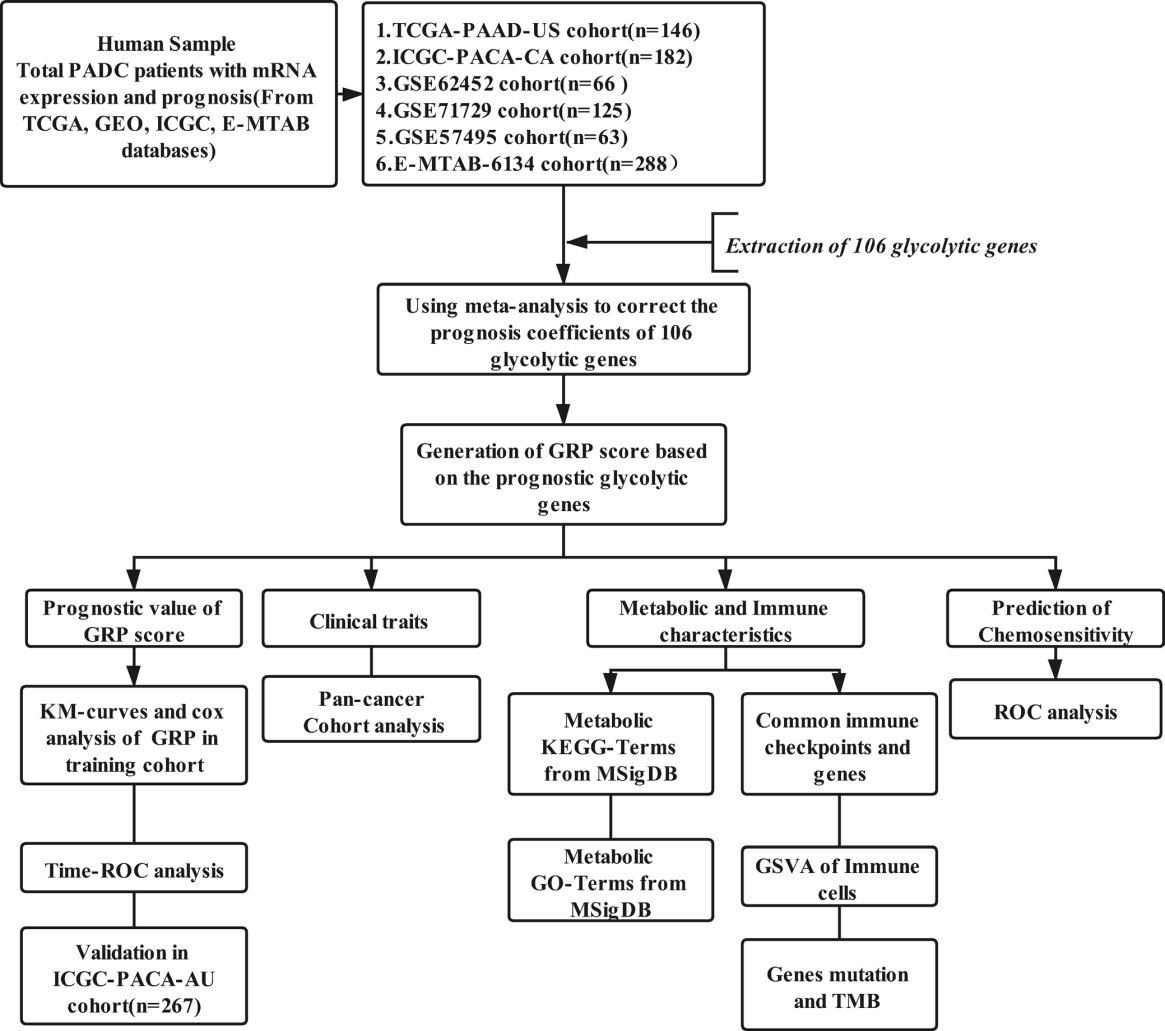
The Flowchart of the present study in the website (https://www.processon.com/diagrams)

The PDAC transcriptome profiles with clinical data were obtained from the TCGA (TCGA, https://portal.gdc.cancer.gov), ICGC (https://dcc.icgc.org/), Arrayexpress (https://ebi.ac.uk/arrayexpress/) and GEO (GEO, http:// www. ncbi.nlm.nih.gov/geo) databases using the following selection criteria for databases: (a): with overall survival (OS) and survival status; (b) large sample size (>50). After filtering, 1024 patients with PDAC from seven data sets were enrolled in this study. Patients from the TCGA‐PAAD‐US (n = 146), ICGC‐PACA‐CA (n = 182), E‐MTAB‐6134(n = 288), GSE71729 (n = 125), GSE57495 (n = 63) and GSE62452 (n = 66) were enrolled to form the training cohort while patients from the ICGC‐PACA‐AU (n = 267) was used as the external validation cohort (Table S1). Although heterogeneity of patients with pancreatic cancer existed, there were no significant differences in overall survival outcomes among the 6 data sets which formed the training cohort and the one data set which formed the validation cohort (Figure 1). The corresponding somatic mutation data of the TCGA and two ICGC‐PACA cohorts were also downloaded from the databases. Data were normalized and log2 transformed for analysis in the GEO platform and FPKM for Illumina platform. The pan‐cancer cohorts with transcriptome profiles and prognostic data from the GDC Pan‐Cancer in the UCSC Public Hub were then downloaded for further analysis (https://xenabrowser.net/datapages/). The design chart of the study is showed in Figure 1.

### 2.2 Meta‐analysis of glycolysis‐related genes

The keywords of ‘glycolytic’ or ‘glycolysis’ were used to search the genes related to glycolysis in the MsigDB (https://www.gsea‐msigdb.org/). On using the term ‘GO_GLYCOLYTIC_PROCESS’, 106 glycolysis genes were downloaded to be used for further analysis with the PDAC patients (Table S1). The cox proportional hazards regression analysis was used to analyse the prognostic evaluation of the 106 glycolysis genes. Each of the glycolysis gene was stratified by its median value into two groups in the PADC training cohort. Finally, the pooled hazard ratio (HR) with 95% CI and SE of HR of glycolysis genes was estimated based on the prognostic results of the training cohort by using the fixed‐effects model of the meta‐analysis.

### 2.3 Development of the prognostic glycolysis‐related genes signature

The pooled HRs of the glycolysis‐related genes with their standard estimates (SE) which were significantly related to prognosis were then integrated as the prognostic glycolytic genes weight to generate the GRP model, which refers to the public method.^18^ The advantage of this method was that it could reduce the impact of sample size on the weight of each gene. The GRP score of a sample is given by the following formula:

$$\mathrm{GRP}\;\mathrm{score}=\sum_{i=1}^{n}\frac{\mathrm{HR}i‐1}{SE(HRi)}*gene\left(i\right)$$

where gene(i) was the relative expression of OS‐related glycolytic gene, n was the total number of OS‐related glycolytic genes, and HR and SE were the pooled results based on meta‐analysis. The normalized Z‐score was then used to calculate the score.

### 2.4 PPI network, immune infiltrating cells, functional and pathway enrichment analysis

The PPI network of potential glycolysis prognostic‐related genes was constructed by using the STRING (http://string‐db.org/). The gene set enrichment analysis (GSEA) was performed to determine whether there was a significant difference in the glycolysis‐related gene sets between the high‐ and low‐GRP scores by using ‘GSVA’ R package for all the PDAC cohorts. In addition, a method of gene set enrichment analysis (GSEA) using a total of 782 marker genes (Table S2) has been proved to be effective to assess the tumour infiltration of 28 immune cell types.^19^ Similarly, the metabolism‐related KEGG and GO terms from the ‘msigdb.v7.0.symbols’ in the MSigDB among the different GRP subgroups were also identified by running the ‘pathifier’ R package in the TCGA transcripts.

### 2.5 Statistical analysis

Statistical analysis of all the clinical data was performed in R 3.6.2. Standard tests included the Student's *t* test, Wilcoxon rank‐sum test and Fisher exact test. The method of Benjamini‐Hochberg (FDR) was used to adjust the *P*‐values for multiple comparisons. The relationship between the GRP Model and other continuous variables was calculated by the Spearman method. The log‐rank test, univariate and multivariate cox proportional hazard regression were used to analyse any related independent predictors of prognosis in PDAC. Time ROC was used to detect the prognostic value of GRP for PDAC patients. The area under the receiver operating characteristic curve (ROC) was used to detect the diagnostic value of GRP for chemosensitivity. All reported *P*‐values were 2‐sided, and statistical significance was set at .05.

## 3 RESULTS

### 3.1 Meta‐analysis to correlate glycolytic genes with prognosis of PDAC patients

**FIGURE 2:**
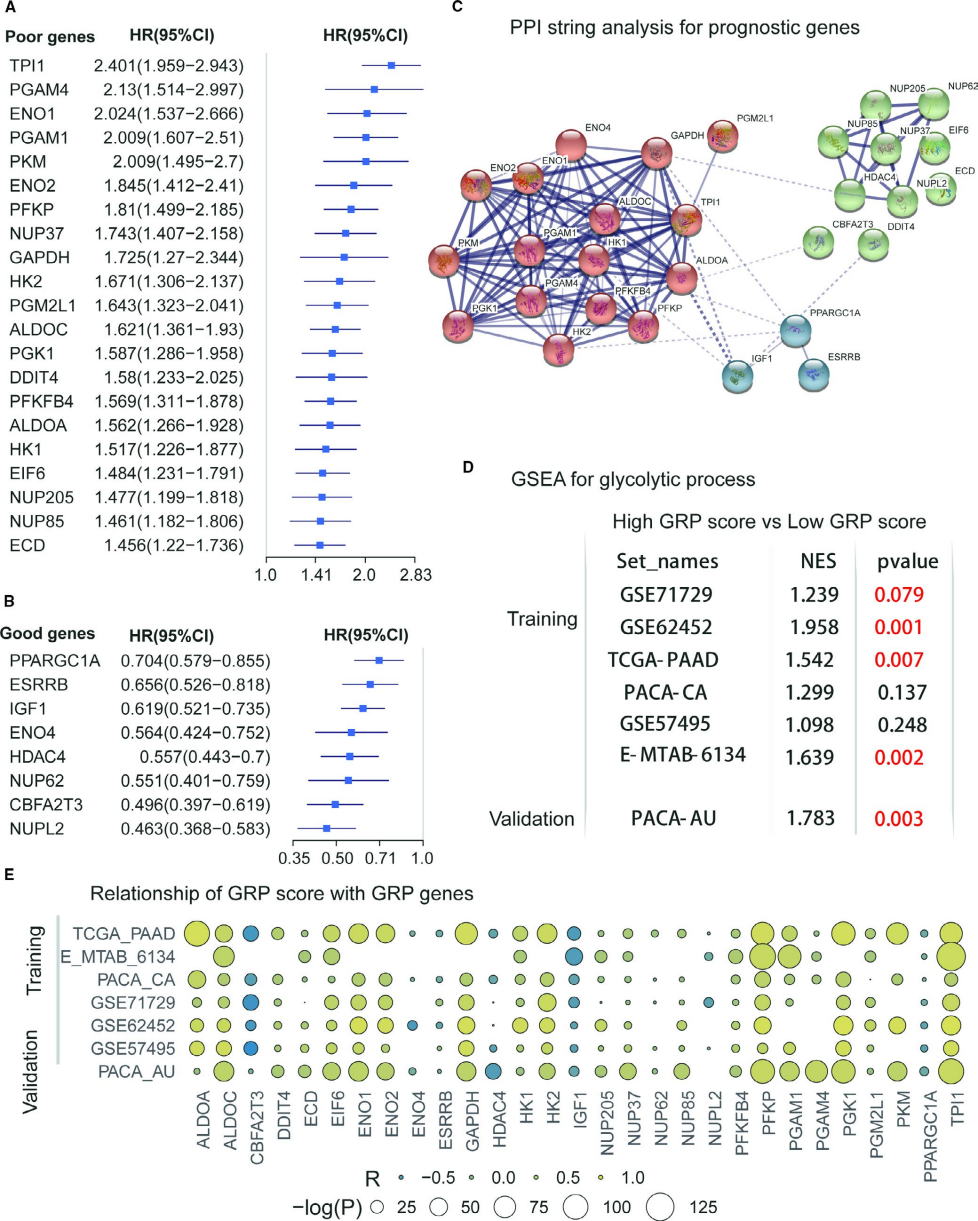
ConstructionoftheGRPscore.(AandB)theprognosticvalueofincludingGlycolyticgenes; (C) PPI analysis in a String of 29 prognostic glycolytic Genes; (D) GSEA analysis for GO_GLYCOLYTIC_PROCESS for the included data sets; (E) association of the GRP score with the 29 prognostic glycolytic genes

With extraction of glycolytic gene expressions from the training and validation cohorts, the cox proportional hazard regression was performed to study the relationship of glycolytic genes with prognosis of PDAC patients (details in Table S1). To obtain the stable and pooled HR and the coefficients of these glycolytic genes in PDAC patients, a meta‐analysis was conducted on the 6 data sets which formed the training cohort. The p‐values of the glycolytic genes were corrected by FDR. The glycolytic genes with significant prognosis were mainly selected based on the following criteria: meta‐analysis, *P* < .001 and FDR < 0.001(Table S3). Of the 29 glycolytic genes which were found to be related to prognosis of PDAC patients, 21 were poor prognosis‐related genes, and 8 were good prognosis‐related genes. The forest plots of 29 glycolytic genes (in Figure 2A,B) show the pooled HRs and 95% CI on meta‐analysis. The PPI network of the 29 glycolytic genes could be separated into three parts (Figure 2C).

### 3.2 Establishing the glycolysis and prognosis‐related genes (GRP) signature

By using the above formula, the GRP score was established by integrating 29 prognostic glycolytic genes. To observe the effect of the GRP score on PDAC patients, patients were divided into the high‐ and low‐glycolysis groups by using the median value of the GRP score in each PDAC cohort. Gene set enrichment analysis (GSEA) showed that patients with PDAC with a high‐GRP score had a stronger active glycolysis process in 5 of 7 data sets compared with patients with PDAC with a low‐GRP score (Figure 2D and Table S4). A lot of significant correlations of the GRP score with most of these included glycolytic genes could be observed in all the data sets (Figure 2E).

### 3.3 Prognostic evaluation of GRP signature in the training and validation cohorts

**FIGURE 3:**
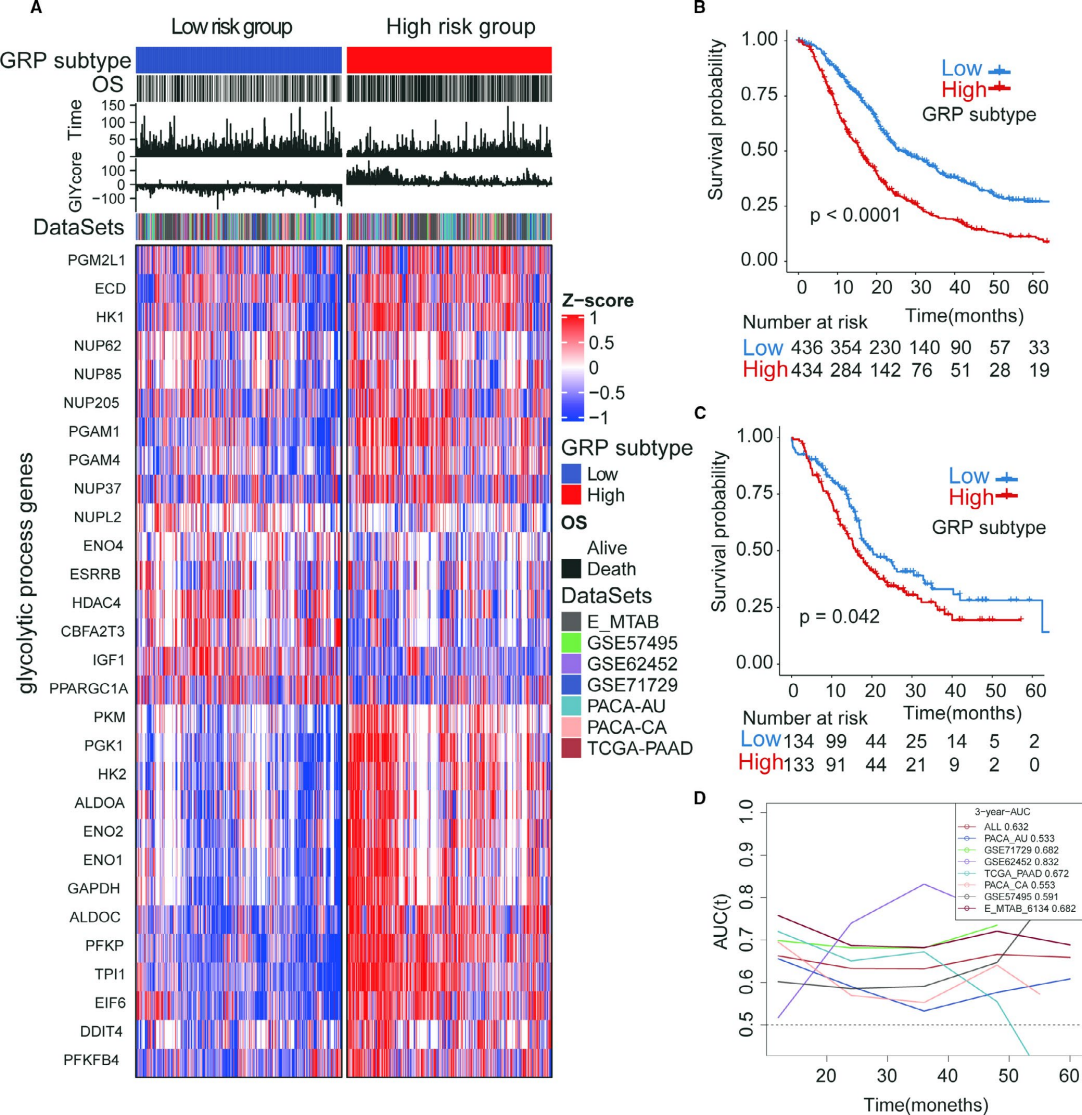
The prognostic value of the GRP score/subtype in the training cohort and validation cohort. (A) heatmap of the 29 prognostic glycolytic genes in the high‐ and low‐GRP subtypes; (B) the Kaplan‐Meier curves of GRP in the training cohort (total patients, n = 1024); (C) the Kaplan‐Meier curves of GRP in the validation cohort (number of patients, n = 267); (D) the time‐dependent ROC of GPR in the training cohort and validation cohort

**FIGURE 4:**
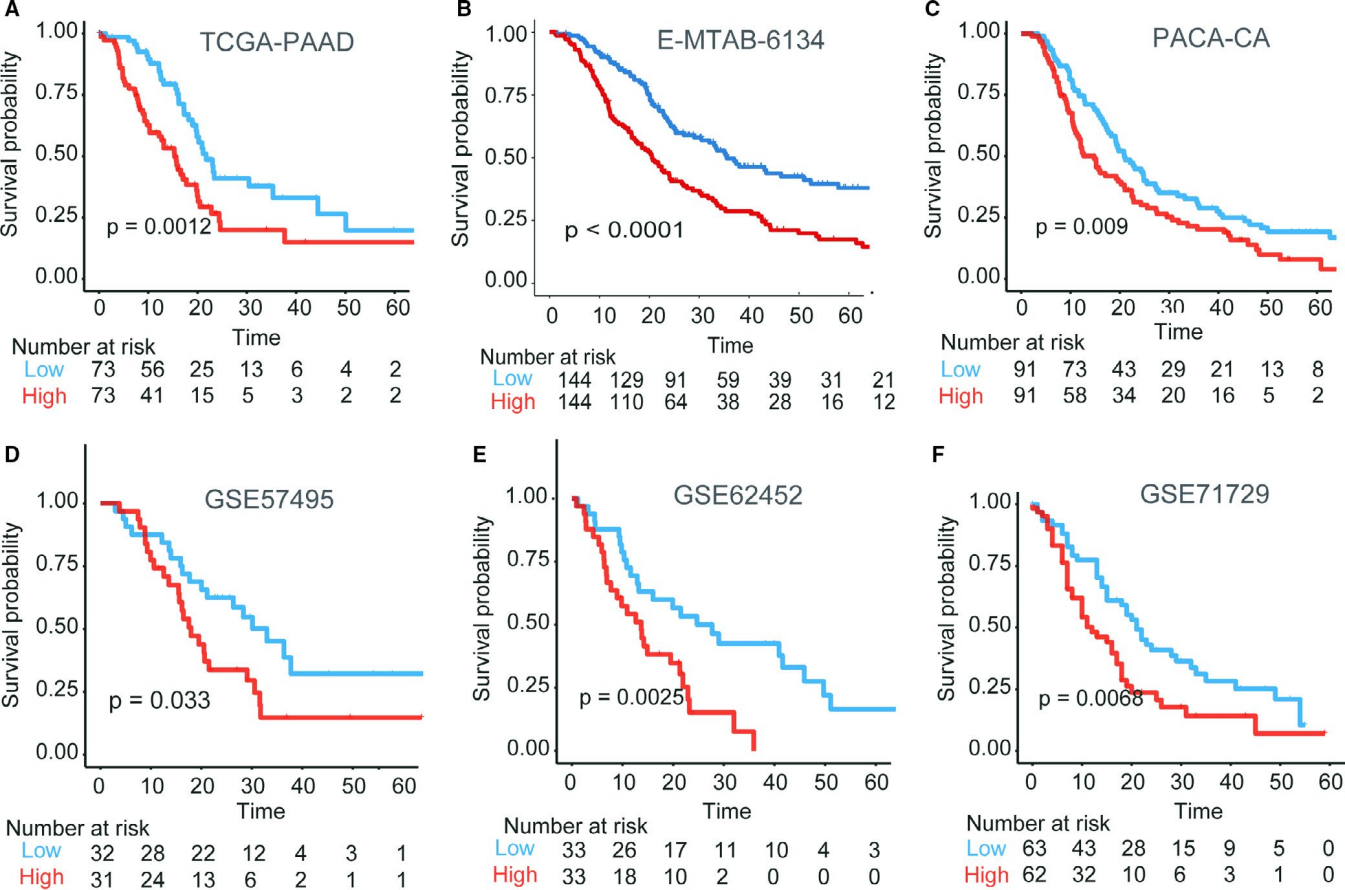
Prognostic evaluation of the GRP subtype in the 6 training data sets. (A) TCGA‐PAAD, high vs low, log‐rank test *P* =.0012; (B) E‐MTAB‐6134, high vs low, log‐rank test *P <* .0001; (C) PCAC‐CA, high vs low, log‐rank test *P* = .009; (D) GSE57495, high vs low, log‐rank test *P* = .033; (E) GSE62452, high vs low, log‐rank test *P* = .0025; (F) GSE71729, high vs low, log‐rank test *P* = .0068

The heatmap of relationships of GRP score with 29 glycolytic genes in all the seven PDAC data sets is showed in Figure 3A. The cut‐off values of the GRP score were truncated by the median values of GRP score in each cohort (Table S5). PDAC patients with high‐GRP scores had worse overall survival outcomes than those patients with low‐GRP scores in the training cohort (Figure 3B, log‐rank test, *P* < .001). This finding was validated in the external validation data set from a different research centre (Figure 3C, log‐rank test, ICGC‐PACA‐AU, *P* < .001). Time‐dependent ROC analysis showed that the GRP signature also had good prediction of OS for PDAC patients in the combination data sets (six training cohorts) (Figure 3D, AUC: range from 0.553 to 0.682, all *P* < .05, and Table S6). In addition, KM curves also showed that the GRP score had a robust ability to distinguish prognosis of PDAC patients in six training cohorts (Figure 4A‐4F, all log‐rank test *P* < .05). Also, the cox regression analysis confirmed that the GRP scores were significantly related to OS in the six data sets and the validation cohort (Table S5, all cox regression *P* < .05). Univariate and multivariate analyses for OS in the TCGA data set showed the GRP score to be an independent risk factor of OS for PDAC patients (Table S7, HR = 1.948, 95%CI = 1.057‐3.588, *P* = .032). As a supplement, we also found that the GRP score could significantly identify differences in recurrence‐free survival for PDAC patients in 2 of the 3 data sets (Figure S2, TCGA‐PAAD and ICGC‐PACA‐AU, log‐rank test *P* < .05).

### 3.4 Clinical and metabolic characteristics of the two GRP score groups

**FIGURE 5:**
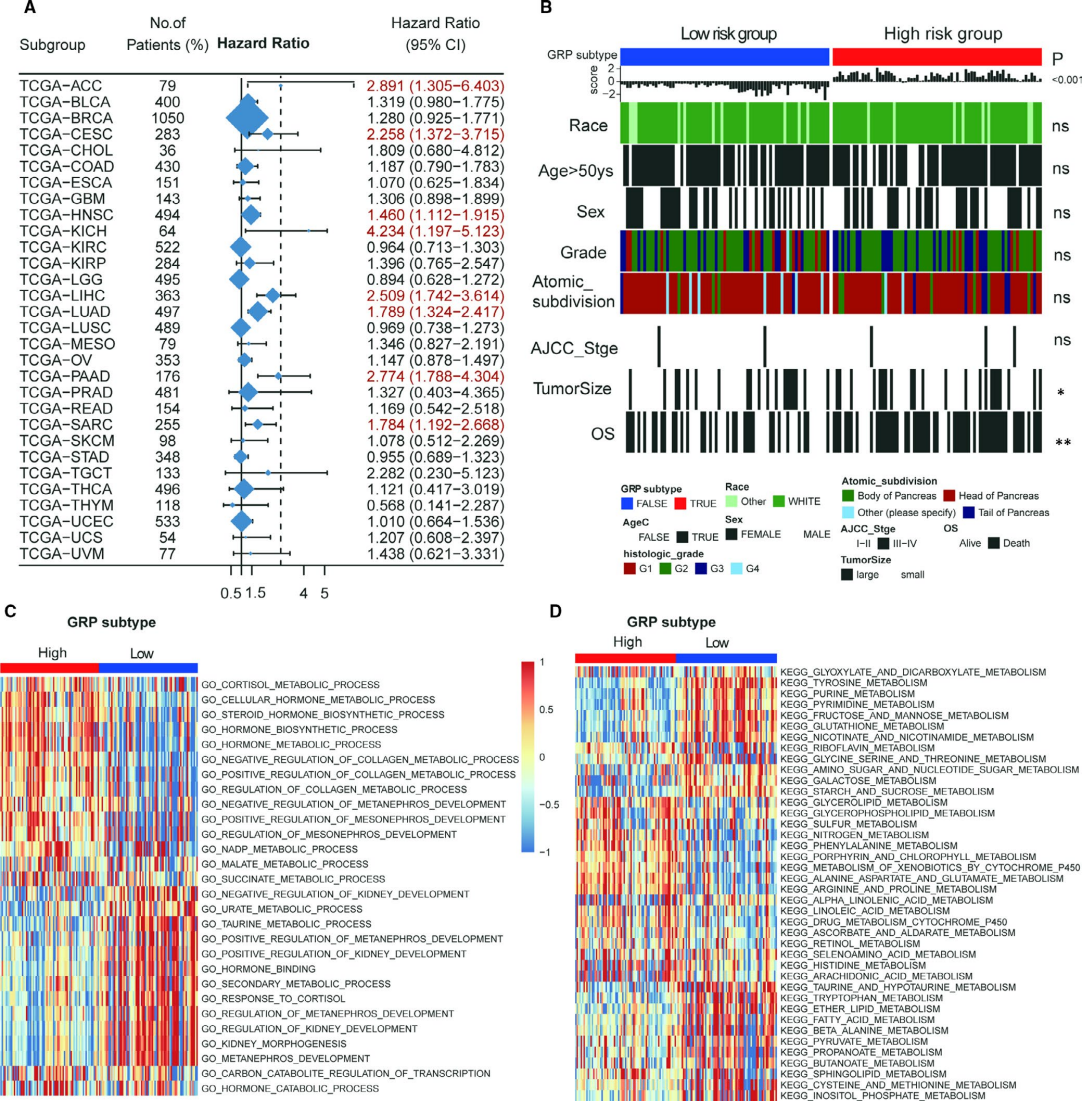
The Association of the GRP score with clinical traits and metabolism pathways in the TCGA data set. A, the prognostic values of the GRP score in the Pan‐cancer data sets (total 30 TCGA data sets). B, the Association of the GRP score with clinical traits in the TGCA data set; (C and D): KEGG and GO terms analysis for the GRP score in the TGCA data set

To evaluate whether the prognostic value of the GRP signature could be generalized, the TCGA pan‐cancer cohorts were further evaluated, and including TCGA‐PAAD cohort, the prognosis of other 7 out of 30 cancers could be significantly distinguished by the GRP score (Figure 5A). In addition to OS, tumour size was positively correlated with the GRP score in PDAC patients in the TCGA cohort (Figure 5B, chi‐square test, *P* < .05). As the glucose metabolism pathway is associated with many other metabolism pathways in vivo, the relationship of the GRP score with the metabolism‐related terms was further explored in the MsigDB. The results showed that 29 GO‐metabolism (Figure 5C) and 40 KEGG‐metabolism gene sets (Figure 5D) were related to the GRP score in the TCGA data set (Table S8).

### 3.5 Relationship of immune components with GRP score

**FIGURE 6:**
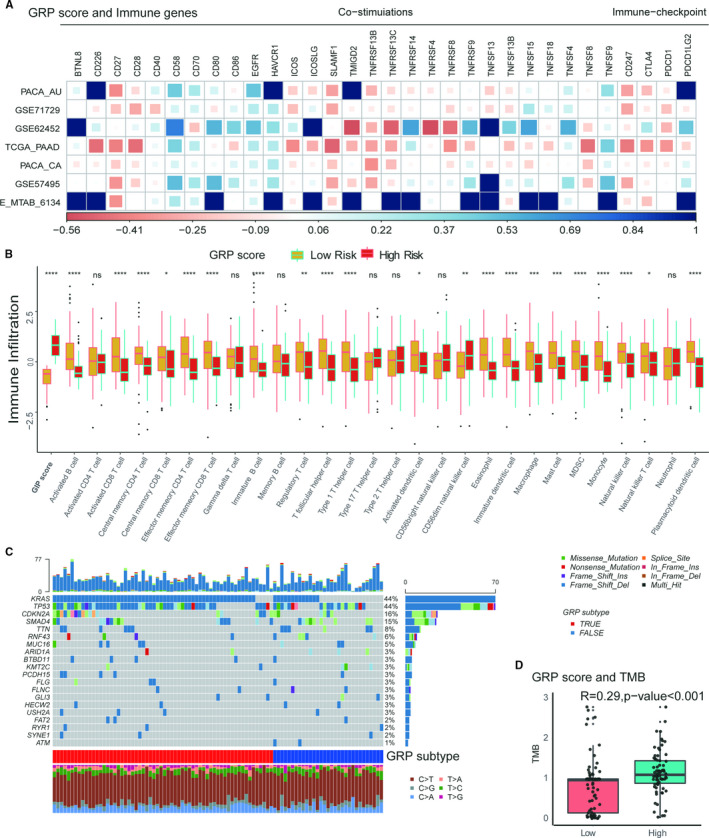
The Associations of the GRP score with immune‐related components in PDAC patients. A, the relationship of the GRP score with immune co‐stimulation and check‐point genes in the total data sets; (B) the different expressions of 29 immune cells (generated by GSVA with 782 immune‐related genes in the high‐ or low‐GRP subtypes; (C) the landscape of top 20‐gene mutations in the TCGA data set. D, the association of TMB with the GRP score)

As metabolic reprogramming has emerged as a crucial player in cancer progression, it becomes important to understand how this metabolic change impacts immune functions. The selected immune genes based on previous studies were used to study the relationship of the GRP score with immune functions. However, there was a huge heterogeneity in the relationship of the GRP score with immune genes in these data sets (Figure 6A). Further investigations on immune cells and gene mutations were then performed in the TCGA data set using gene set enrichment analysis (GSEA). The GSVA R package was used to generate infiltration of 28 immune cells in 782 immune genes. Most of these immune cells were decreased in the high‐GRP group and increased in the low‐GRP group (Figure 6B, Mann‐Whitney U, *P* < .05) indicating that activation of the glycolysis process reduced infiltration of immune cells in PDAC patients (Figure 6B). Oncoplot also showed the most frequent genes mutations in PDAC to include KRAS, TP53, CDKN2A and SMAD4 (Figure 6C). Only KRAS had a significantly higher frequency in the high than the low‐GRP groups (Figure 6C, chi‐square test, *P* < .001). However, the tumour mutation burden (TMB) of PDAC patients were still significantly different between the two GRP groups (Figure 6D, Mann‐Whitney U, *P* < .001). Similar results were obtained in the ICGC‐PACA‐AU and ICGC‐PACA‐CA data sets (Figure S3).

### 3.6 Prediction of the GRP score on chemosensitivity

**FIGURE 7:**
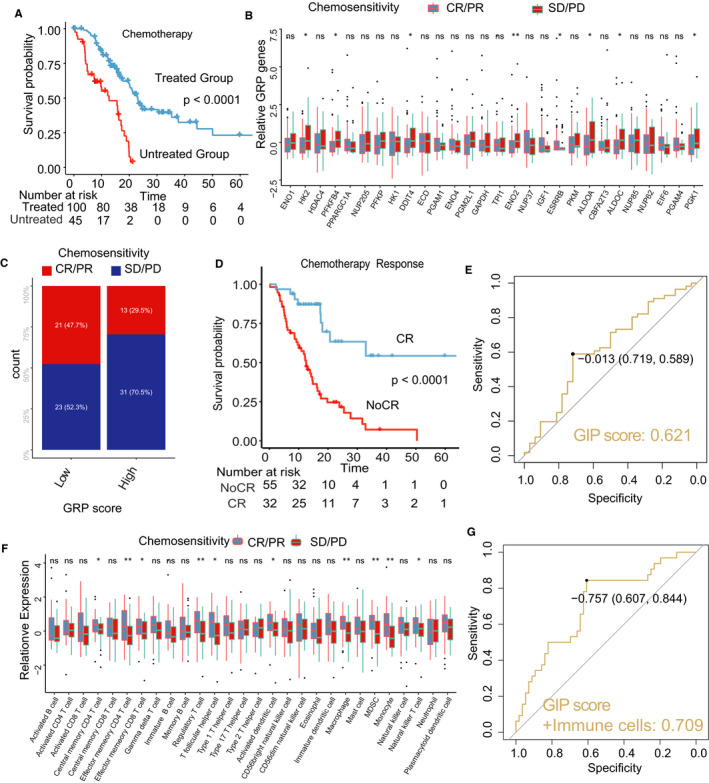
The predicted evaluation of the GRP score in chemotherapy. A, Kaplan‐curves of chemotherapy vs non‐chemotherapy; (B) the different expressions of GRP genes in the high‐ or low‐GRP groups; (C) Chi‐square test for the GRP score and response of chemotherapy; (D) Kaplan‐curves of CR (complete response) vs Non‐CR in patients with chemotherapy vs non‐chemotherapy; (E) the diagnostic evaluation of the GRP score for (TCGA data set). F, the relative expressions of 28 immune cells in the high‐ or low‐GRP groups; (G) the combination of the GRP score and immune cells in diagnosis of CR/PR to chemotherapy

As PDAC patients who underwent chemotherapy obtained better overall survival outcomes (Figure 7A, log‐rank test< 0.001), the differences for the 29 glycolysis genes between the CR/PR and PD/SD groups were tested (Figure 7B). The glycolysis genes, including HK2, PFKFB4, DDIT4 ENO2, ESRRB, ALDOC and PGK1 genes (Figure 7B, P < .05, TCGA data set), and the GRP score (Figure 7C, Mann‐Whitney U, *P* < .001) were significantly related to chemosensitivity in PDAC patients. The PDAC patients with CR had a significant better OS than those with No‐CR (Figure 7D, log‐rank test, *P* < .001). ROC analysis showed that the GRP score predicted CR in patients with PDAC who underwent chemotherapy (Figure 7E, AUC = 0.621, *P* < .001). Similar results were obtained in the other ICGC‐PACA‐CA data sets (Figure S4). As previous studies have shown infiltration of immune cells to be related to chemosensitivity responses in cancers, further analysis was carried out which showed that 11 of 28 immune cells were significantly related to chemosensitivity in PDAC patients (Figure 7F, TCGA cohort, *P* < .05). Finally, good prediction for CR was obtained by combining the GRP score with immune cells (Figure 7G, AUC = 0.709).

## 4 DISCUSSION

Cancer cell metabolism, as a hallmark of cancer cells, has recently attracted enormous interest along with the parallel explosion of genomic, transcriptomic, proteomic and epigenetic profiling of cancers.^20^ Metabolism of malignant tumours is characterized by the Warburg effect, which represents aerobic glycolysis and indicates some cancers hold their breath.^21^ Aerobic glycolysis is essential for cancer cells growth and invasion.^22^, ^23^, ^24^ In PDAC, recent studies reported that aerobic glycolysis of metabolic reprogramming promoted pancreatic tumorigenesis, proliferation and metastasis.^8^, ^25^, ^26^, ^27^ Although there are many studies on the relationship between PDAC and glycolysis, researches which involve biomarkers and prognosis of patients with PDAC relating to glycolysis are seldomly reported.

This study focused on the association between genes relating to the glycolysis pathway and prognosis in patients with PDAC. It identified glycolysis‐related biomarkers for patients with PDAC and established a scoring system to distinguish groups of PDAC patients with different prognosis and sensitivity to chemotherapy. In this study, a GRP score was obtained from PPI analysis on 29 prognostic glycolytic genes, which distinguished PDAC patients with different overall survival outcomes in the training cohort of 1024 patients from 6 data sets and a validation cohort of 267 patients from a different data set. The relationship between the GRP score with the immune‐related components, including immune co‐stimulation, check‐point genes and TMB, in PDAC was uncovered. The highlights of the results of this study are that the GRP score could be used to predict sensitivity and response of PDAC to chemotherapy, and by combining with immune‐related cells in predicting complete or partial response of PDAC to chemotherapy.

An accurate prognostic assessment of cancers could help clinicians to make appropriate treatment decisions.^28^ Precision therapy based on molecular biomarkers has improved prognostic estimates for patients with PDAC.^29^, ^30^ Previous studies reported a disease‐related single nucleotide polymorphism (SNP)‐based genetic risk score could provide independent information on PDAC risk and could be used to predict high‐risk patients in a PDAC population.^31^ Another polygenic and multifactorial score could be applied for pancreatic ductal adenocarcinoma risk prediction.^32^ To better prognosticate patients with PDAC, researchers have established a 20‐gene score by utilizing publicly available high‐throughput transcriptomic data from GEO, TCGA and ICGC which have also reported on OS data. This 20‐gene pancreatic cancer prognostic score could define not only prognostic and biological subgroups, but predicted their responses to targeted therapy.^33^ However, these risk scores did not focus on genes relating to glucose metabolism in PDAC, and the prediction efficiency was relatively low. With the reported vital role of Warburg effect in PDAC, the current study screened out prognostic glycolytic genes relating to PDAC and developed a GRP score to predict prognosis and survival risks in patients with PDAC. A previous study identified novel genes associated with poor prognosis in pancreatic ductal adenocarcinoma by carrying bioinformatics analysis on PKM and PPARG.^34^ The current study, by screening similar prognostic glycolytic genes as in the previous study,^34^ also found PKM2 to promote pancreatic ductal adenocarcinoma invasion and metastasis.^12^ In addition, altering expression levels of HK and PKM2 with metabolic inhibitors showed favourable effects on PDAC, thus identifying these as potential therapeutic targets^35^ as these glycolytic genes play an imperative role in PDAC. Importantly, this current study is the first study to integrate 29 prognostic glycolytic genes in establishing a GRP score, which distinguished patients with PDAC into high‐ and low‐GRP subgroups with different survival outcomes.

Tumour immunity has provided not only a new perspective for tumour treatment, but also improves prognosis by combining chemotherapy and immunotherapy.^36^ PDAC exhibits an immunosuppressive microenvironment. As immune response predicts survival, activation of the immune system has the potential to produce an efficacious PDAC therapy.^37^ Immune cells can have an impact on the composition of pancreatic stroma to affect progression of PDAC.^38^ Recent studies indicated that the hexosamine biosynthesis pathway (HBP), which is a shunt pathway of glycolysis, is a metabolic node in cancer cells that can promote survival pathways on one hand, and influence hyaluronan synthesis in the extracellular material (ECM) on the other. Researchers who targeted glutamine‐fructose amidotransferase 1 (GFAT1) of the rate‐limiting enzyme of this pathway by using a small molecule glutamine analog (6‐diazo‐5‐oxo‐l‐norleucine), could sensitize pancreatic tumours to anti‐PD1 therapy, thus, resulted in tumour regression and prolonged survival.^39^

In addition, previous studies have proved that the glycolysis process can interfere antitumorigenic functions of immune cells and achieve immune evasion in cancers.^40^, ^41^, ^42^ The relationship between glycolysis metabolism and infiltration immune cells in PDAC has never been explored. In our study, we found immune cells were decreased in the high‐GRP group, and increased in the low‐GRP group, so we speculated that the decrease of infiltrating immune cells might be associated with the glycolysis process. Recently, a few studies have revealed the relationship between the glycolysis and infiltrating immune cells. For example, Li et al explored the relationship between tumour glycolysis and immune function in breast cancer using the TCGA data set. They found that breast cancer patients in high‐glycolysis group had a lower infiltration of tumour‐killing immune cells such as NKT cells, CD8+ T cells, CD8+ Tcm and cDC cells.^43^ Moreover, a proteogenomic study on colon cancer found that in microsatellite instability‐high (MSI‐H) type of colon cancer, increased glycolysis was associated with decreased CD8 T cell infiltration.^44^ The author also mentioned and endorsed a 2018 study,^45^ which reported that increased tumour glycolysis suppresses anti‐tumour immunity by impairing T cell function and trafficking to the tumour microenvironment. In addition, in 2018, Cascone et al proved that melanoma and lung cancer patients with lower infiltrated T cells had high‐expressed glycolysis‐related genes.^46^ They thought that tumour glycolytic activity was negatively correlated with tumour infiltration of T cells in those two diseases. As outlined above, presently, only a few studies have reported the relationship between the process of glycolysis in several kinds of cancers and tumour immune cell infiltration. We think this is an interesting phenomenon that deserves further investigation. In the future, more exploration needs to be conducted to determine the relationship between the process of glycolysis and infiltration immune cells in more kinds of cancers. In addition, we found that above researches revealed that tumour glycolysis mainly inhibited T cell infiltration. The relationship between the glycolysis and more types of infiltration immune cells in tumours needs to be further explored.

Chemotherapy is still a first‐line treatment for advanced or metastatic PDAC.^47^ Due to tumour heterogeneity in PDAC, not all PDAC is sensitive to chemotherapy. Recent studies reported that germline variants in human DNA damage repair genes were associated with response to adjuvant chemotherapy after surgical resection for PDAC.^48^ In addition, as the dense hypovascularized stroma in PDAC is widely different from many other solid tumours, the stroma acts as a dominant factor in limiting delivery of almost all drugs to tumour cells, which is a key link in severe drug resistance at the tumour microenvironment level.^49^ Currently, more than 100 genes have been found to be implicated in drug resistance of pancreatic tumours, including RAS and CXCR4.^50^ Moreover, drug resistance in PDAC is thought to be mediated by modulation of miRNAs (eg miRNA‐21), which regulate genes that participate in cell proliferation, invasion and metastasis.^50^ Cancer stem cells are also intimately related to drug resistance in PDAC.^51^ In other words, the mechanisms of drug resistance at the molecular level are vital for further studies. This current study demonstrated that the GRP score predicted sensitivity of chemotherapy (complete or partial response) with an AUC of 0.621, and a combination of the GRP score and immune cells in diagnosing CR/PR to chemotherapy with an AUC of 0.706. Thus, the GRP score, based on genes related to glucose metabolism, not only predicted prognosis of patients with PDAC, but predicted chemosensitivity of PDAC.

There are limitations in this study. First, the prognostic effectiveness of the GRP score in patients with PDAC should further be tested and verified in prospective clinical studies. Second, biological mechanisms by which the candidate markers relating to glycolysis, which contribute to progression and chemoresistance of PDAC remain largely unclear. Further studies into the functions can provide better clues for targets and treatment strategies.

In conclusions, a GRP score relating to glycolysis that can predict survival outcomes of patients with PDAC was identified and verified in this study. Higher risk scores indicated unfavourable survival outcomes. The GRP score, when combined with data on tumour immune cells, could be used in predicting chemosensitivity of PDAC. Novel insights into the relationship between glycolysis and PDAC were shown in this study, and the glycolysis‐related genes in the GRP score were shown to be promising prognostic targets in future clinical studies, and in identifying patients with PDAC with poor prognoses. The results of this study can also be used for future studies on personalized treatment for patients with PDAC.

## CONFLICT OF INTEREST

The authors have declared that no competing interest exists.

## AUTHOR CONTRIBUTIONS

**Xiu‐Ping Zhang:** Investigation (equal); Resources (equal); Validation (equal); Writing‐original draft (equal); Writing‐review & editing (equal). **Qinjunjie Chen:** Data curation (equal); Formal analysis (equal); Methodology (equal); Resources (equal); Software (equal). **Qu Liu:** Data curation (equal); Formal analysis (equal); Resources (equal); Software (equal); Validation (equal). **Yang Wang:** Conceptualization (equal); Investigation (equal); Methodology (equal); Resources (equal); Validation (equal). **Fei Wang:** Data curation (equal); Formal analysis (equal); Methodology (equal); Resources (equal). **Zhi‐Ming Zhao:** Conceptualization (equal); Data curation (equal); Methodology (equal); Visualization (equal). **Guo‐Dong Zhao:** Resources (equal); Software (equal); Validation (equal); Visualization (equal). **Wan Yee Lau:** Conceptualization (equal); Data curation (equal); Project administration (equal); Supervision (equal); Writing‐original draft (equal); Writing‐review & editing (equal). **Yu‐Zhen Gao:** Conceptualization (equal); Data curation (equal); Formal analysis (equal); Investigation (equal); Methodology (equal); Resources (equal); Software (equal); Validation (equal); Visualization (equal). **Rong Liu:** Conceptualization (lead); Project administration (lead); Resources (lead); Supervision (lead).

## Supporting information

Fig S1‐S4

Table S1‐S8

## Data Availability

All data generated or analysed during this study are freely available in the published articles and the public research databases, which are open source.
