# Supplementary material for: Development and validation of glycolysis‐related prognostic score for prediction of prognosis and chemosensitivity of pancreatic ductal adenocarcinoma
Source: J Cell Mol Med. 2021 May 3;25(12):5615–27. doi: 10.1111/jcmm.16573 (PMC8184720; doi:10.1111/jcmm.16573)
Supplement: Supplementary file 1 — Fig S1‐S4 [file JCMM-25-5615-s001.docx]

**Supplemental Figures for the manuscript entitled “Development and Validation of Glycolysis-related Prognostic Score for Prediction of Prognosis and Chemosensitivity of Pancreatic Ductal Adenocarcinoma”**


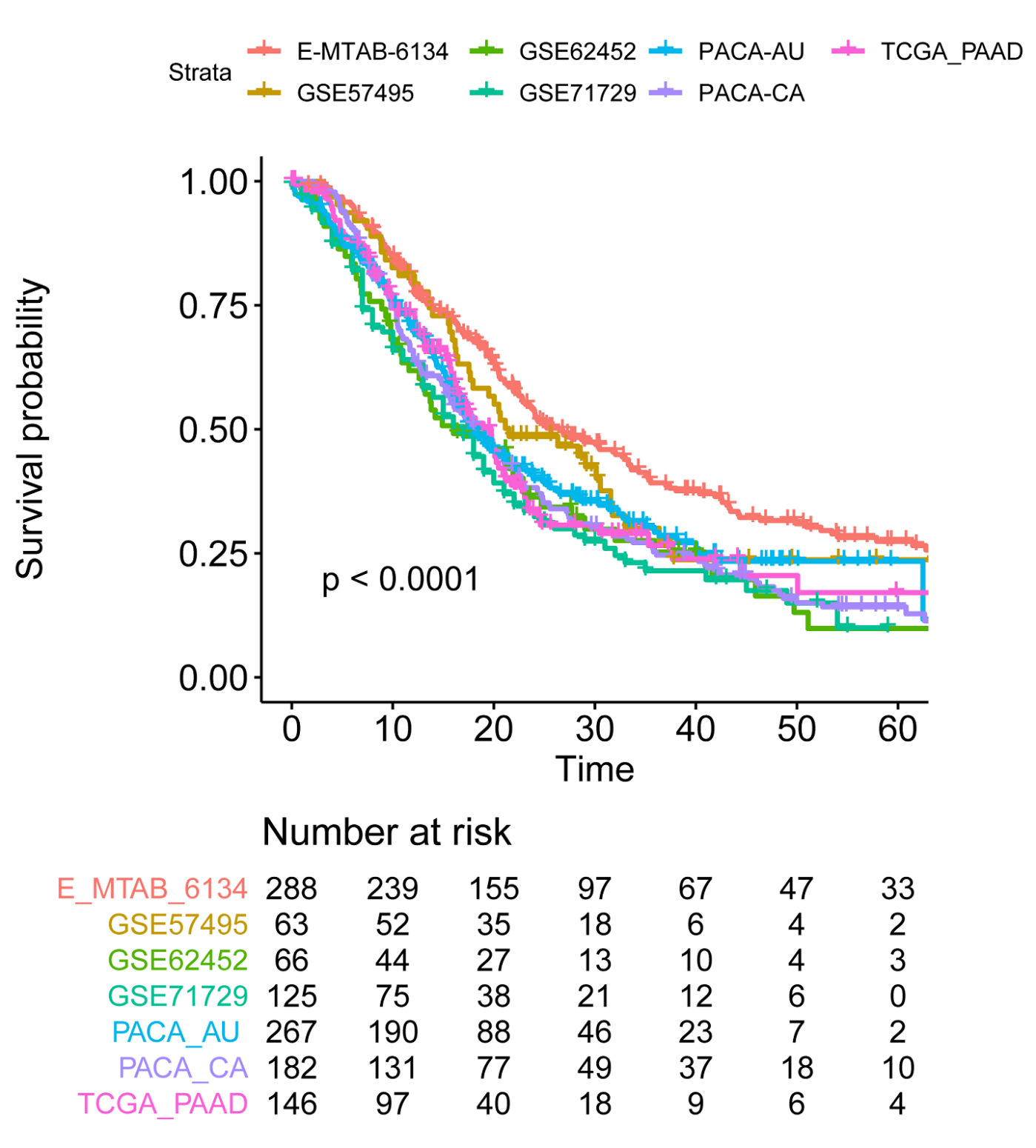


**Figure S1. KM curves of OS for all cohorts**


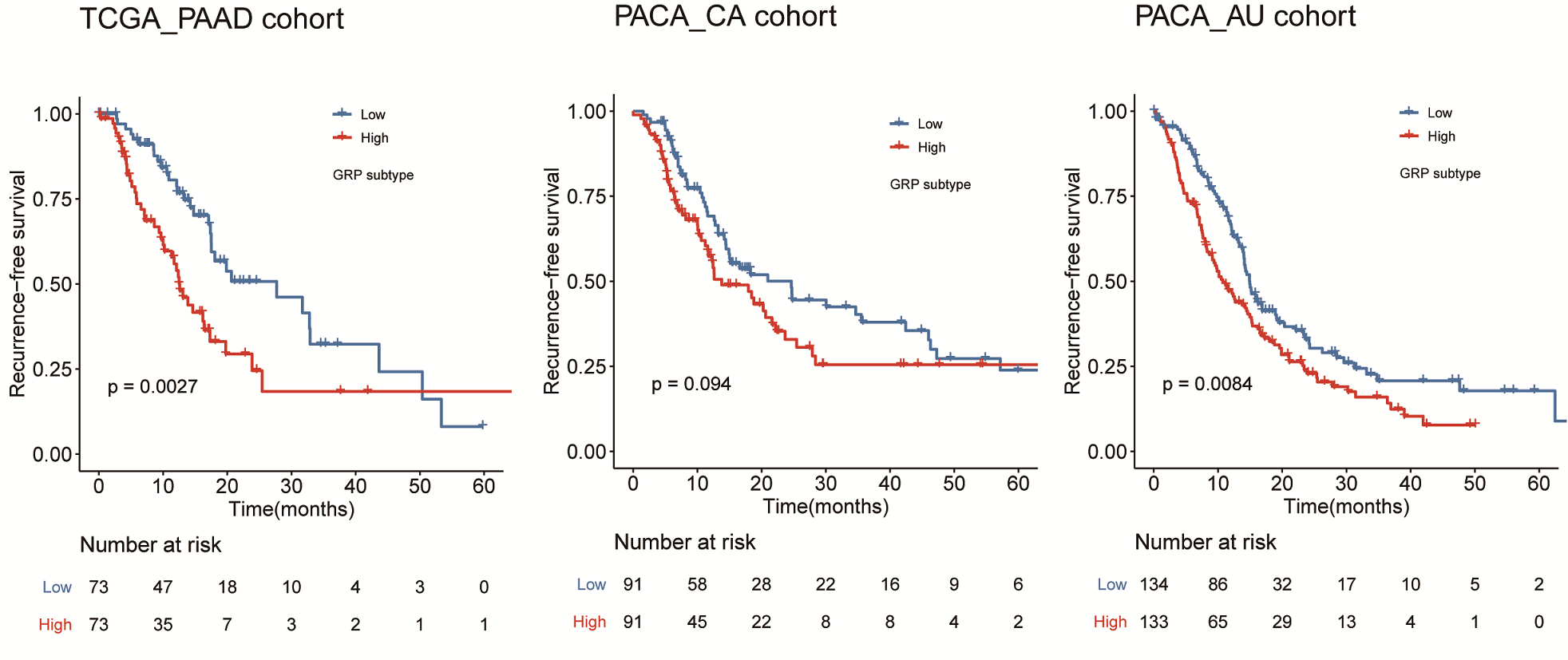


**Figure S2. The KM curves of RFS among different GRP score groups in three cohorts**


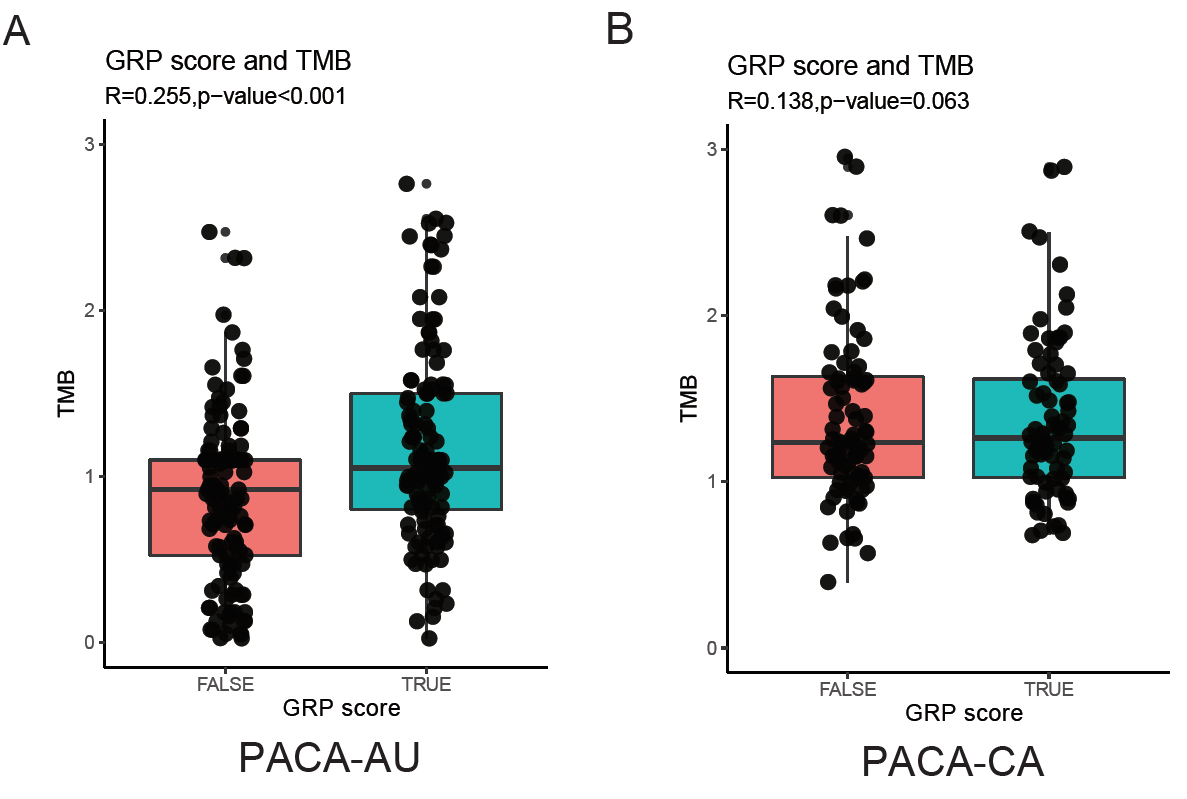


**Figure S3. The distribution of TMB in different GRP score groups**


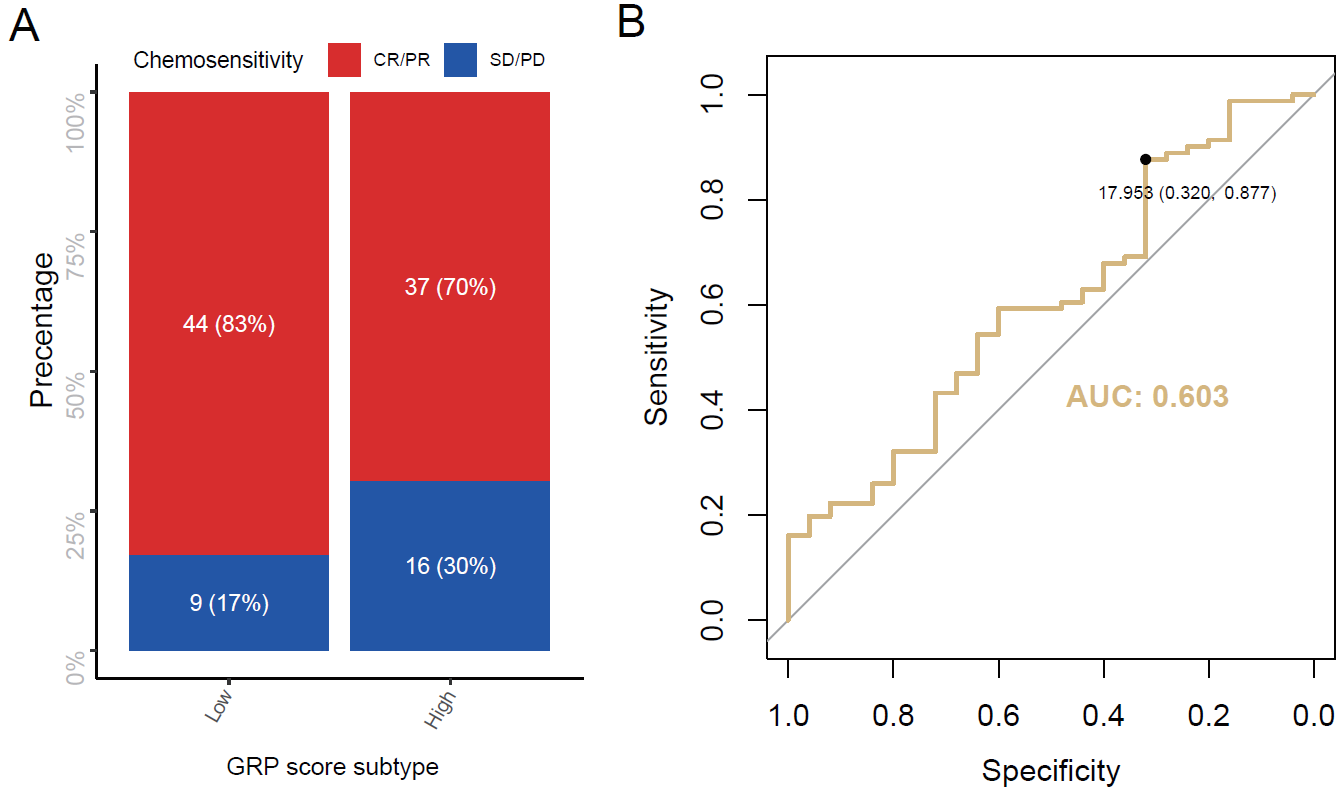


**Figure S4. The percentages of chemotherapy reaction (CR/PR vs. SD/PD) in different GRP score groups (A), and the ROC curves of CR/PR after chemotherapy in PDAC patients (B).**
